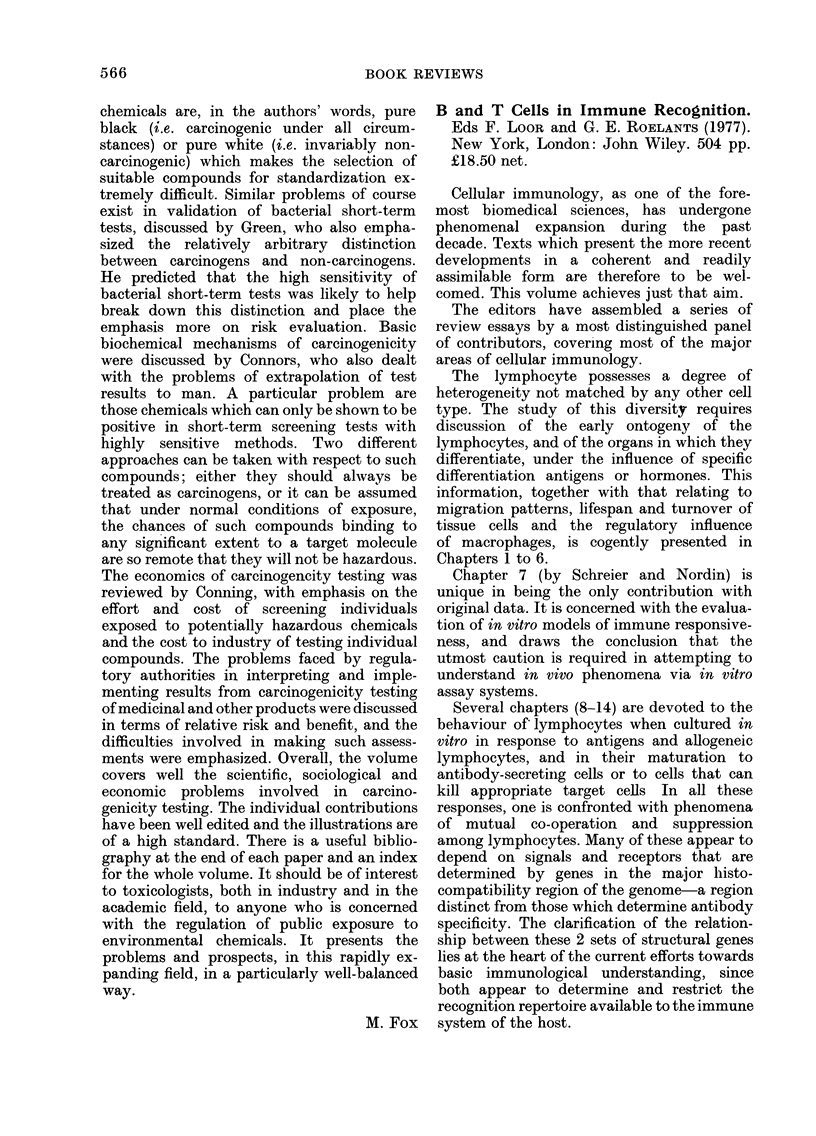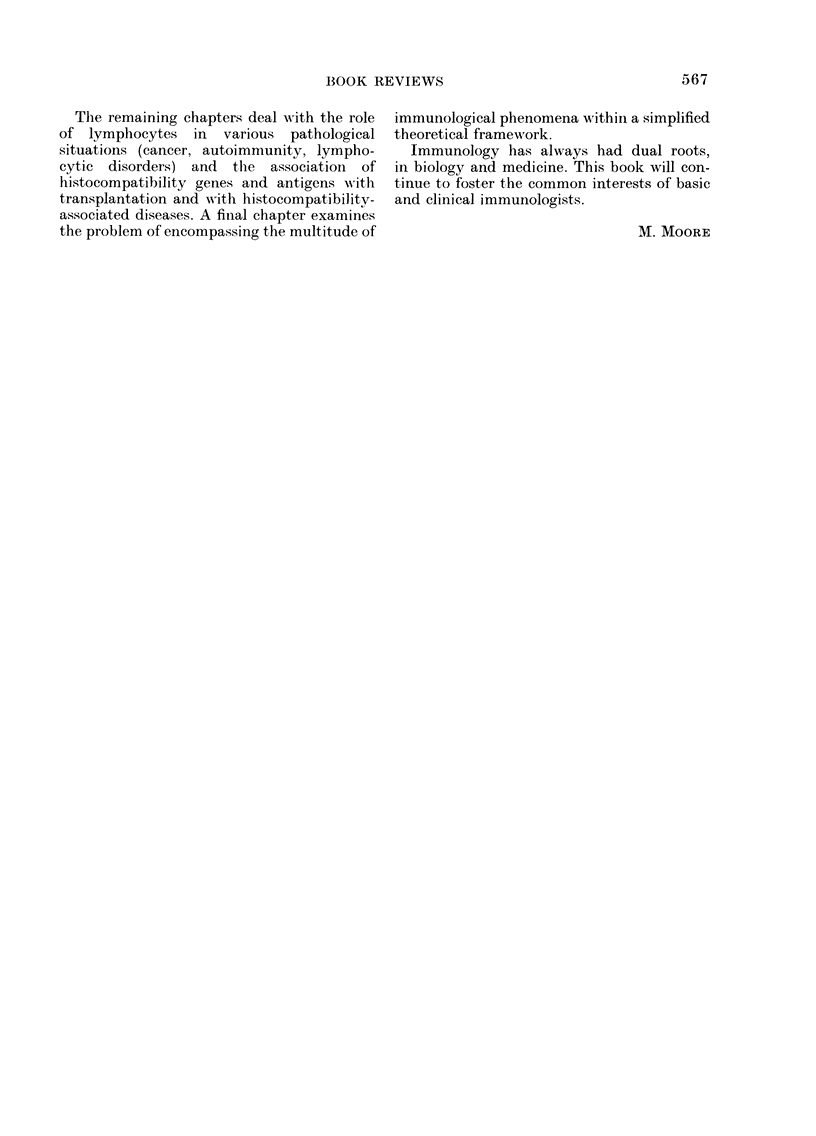# B and T Cells in Immune Recognition

**Published:** 1978-10

**Authors:** M. Moore


**B and T Cells in Immune Recognition.** Eds F. Loor and G. E. Roelants (1977). New York, London: John Wiley. 504 pp. £18.50 net.

Cellular immunology, as one of the foremost biomedical sciences, has undergone phenomenal expansion during the past decade. Texts which present the more recent developments in a coherent and readily assimilable form are therefore to be welcomed. This volume achieves just that aim.

The editors have assembled a series of review essays by a most distinguished panel of contributors, covering most of the major areas of cellular immunology.

The lymphocyte possesses a degree of heterogeneity not matched by any other cell type. The study of this diversity requires discussion of the early ontogeny of the lymphocytes, and of the organs in which they differentiate, under the influence of specific differentiation antigens or hormones. This information, together with that relating to migration patterns, lifespan and turnover of tissue cells and the regulatory influence of macrophages, is cogently presented in Chapters 1 to 6.

Chapter 7 (by Schreier and Nordin) is unique in being the only contribution with original data. It is concerned with the evaluation of *in vitro* models of immune responsiveness, and draws the conclusion that the utmost caution is required in attempting to understand *in vivo* phenomena via *in vitro* assay systems.

Several chapters (8–14) are devoted to the behaviour of lymphocytes when cultured *in vitro* in response to antigens and allogeneic lymphocytes, and in their maturation to antibody-secreting cells or to cells that can kill appropriate target cells In all these responses, one is confronted with phenomena of mutual co-operation and suppression among lymphocytes. Many of these appear to depend on signals and receptors that are determined by genes in the major histocompatibility region of the genome—a region distinct from those which determine antibody specificity. The clarification of the relationship between these 2 sets of structural genes lies at the heart of the current efforts towards basic immunological understanding, since both appear to determine and restrict the recognition repertoire available to the immune system of the host.

The remaining chapters deal with the role of lymphocytes in various pathological situations (cancer, autoimmunity, lymphocytic disorders) and the association of histocompatibility genes and antigens with transplantation and with histocompatibility-associated diseases. A final chapter examines the problem of encompassing the multitude of immunological phenomena within a simplified theoretical framework.

Immunology has always had dual roots, in biology and medicine. This book will continue to foster the common interests of basic and clinical immunologists.

M. MOORE